# Neuroacanthocytosis associated with a defect of the 4.1R membrane protein

**DOI:** 10.1186/1471-2377-7-4

**Published:** 2007-02-13

**Authors:** Antonio Orlacchio, Paolo Calabresi, Adriana Rum, Anna Tarzia, Anna Maria Salvati, Toshitaka Kawarai, Alessandro Stefani, Antonio Pisani, Giorgio Bernardi, Paolo Cianciulli, Patrizia Caprari

**Affiliations:** 1 Laboratorio di Neurogenetica, Centro Europeo di Ricerca sul Cervello (CERC) – Istituto di Ricovero e Cura a Carattere Scientifico (IRCCS) Santa Lucia, Rome, Italy; 2 Dipartimento di Neuroscienze, Neurologia, Università di Roma "Tor Vergata", Rome, Italy; 3 Dipartimento di Specialità Medico-Chirurgiche e Sanità Pubblica, Neurologia, Università di Perugia, Perugia, Italy; 4 Laboratorio di Neurofisiologia Sperimentale, Centro Europeo di Ricerca sul Cervello (CERC) – Istituto di Ricovero e Cura a Carattere Scientifico (IRCCS) Santa Lucia, Rome, Italy; 5 Dipartimento di Ematologia, Oncologia e Medicina Molecolare, Istituto Superiore di Sanità, Rome, Italy; 6 Department of Neurology, Hyogo Brain and Heart Center, Himeji city, Hyogo prefecture, Japan; 7 Day Hospital Talassemici, Ospedale S. Eugenio, Rome, Italy

## Abstract

**Background:**

Neuroacanthocytosis (NA) denotes a heterogeneous group of diseases that are characterized by nervous system abnormalities in association with acanthocytosis in the patients' blood. The 4.1R protein of the erythrocyte membrane is critical for the membrane-associated cytoskeleton structure and in central neurons it regulates the stabilization of AMPA receptors on the neuronal surface at the postsynaptic density. We report clinical, biochemical, and genetic features in four patients from four unrelated families with NA in order to explain the cause of morphological abnormalities and the relationship with neurodegenerative processes.

**Case presentation:**

All patients were characterised by atypical NA with a novel alteration of the erythrocyte membrane: a 4.1R protein deficiency. The 4.1R protein content was significantly lower in patients (3.40 ± 0.42) than in controls (4.41 ± 0.40, *P *< 0.0001), reflecting weakened interactions of the cytoskeleton with the membrane. In patients IV:1 (RM23), IV:3 (RM15), and IV:6 (RM16) the 4.1 deficiency seemed to affect the horizontal interactions of spectrin and an impairment of the dimer self-association into tetramers was detected. In patient IV:1 (RM16) the 4.1 deficiency seemed to affect the skeletal attachment to membrane and the protein band 3 was partially reduced.

**Conclusion:**

A decreased expression pattern of the 4.1R protein was observed in the erythrocytes from patients with atypical NA, which might reflect the expression pattern in the central nervous system, especially basal ganglia, and might lead to dysfunction of AMPA-mediated glutamate transmission.

## Background

Acanthocytosis is found to be associated with at least three hereditary neurological disorders that are generally referred to as neuroacanthocytosis (NA): Chorea-acanthocytosis (ChAc), Abetalipoproteinaemia, and McLeod syndrome [1,2]. ChAc is typically an autosomal recessive condition characterised by chorea, orofaciolingual dyskinesia, dysphagia, dysarthria, areflexia, seizures, and dementia. Abetalipoproteinaemia is also an autosomal recessive condition characterised by the absence of serum apoliprotein B resulting in progressive spinocerebellar ataxia with peripheral neuropathy, chorea, and retinitis pigmentosa. McLeod syndrome is an X-linked disorder characterised by the abnormal expression of the Kell blood group antigen and by elevated serum creatine kinase levels, myopathy, and chorea [1,2].

Abnormalities of the major integral membrane protein band 3 have been reported in cases of autosomal recessive ChAc, although the relationship between this alteration and the neurodegenerative processes has yet to be determined [1].

We performed clinical, biochemical, and genetic investigations in four patients with atypical NA associated with a novel alteration of erythrocyte membrane: that is a defect of the 4.1R protein. Erythroid 4.1R protein is essential for maintaining erythrocyte shape and mechanical properties of the membrane, such as deformability and stability. 4.1R protein stabilizes the spectrin-actin network (horizontal interactions) and mediates the attachment of the underlying cytoskeleton to the overlaying lipid bilayer through vertical interactions with lipids as well as with integral membrane proteins (protein band 3 and glycophorins). Deficiency of 4.1R in red blood cells leads to the assembly of an unstable cytoskeleton structure that manifests itself by the loss of normal discoid morphology [3].

The 4.1R protein is expressed in many tissues [4,5] and in specific neuronal populations, since it is a key element in the regulation of central synapses [6,7]. An association between a defect of 4.1R protein and the expression of hyperkinetic disorders is hypothesised.

This study was approved by the Ethics Committee of the "Istituto di Ricovero e Cura a Caratttere Scientifico" (IRCCS) Santa Lucia, Rome, Italy. Informed consent was obtained from all participants of the pedigrees.

**Figure 1:**
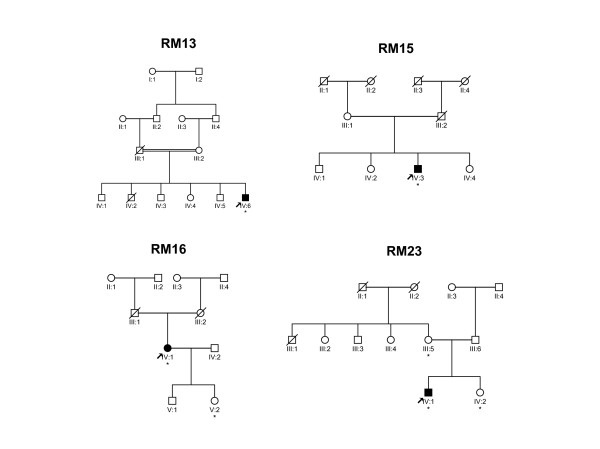
Pedigrees of families RM13, RM15, RM16, and RM23. Black squares (males) and circles (females) indicate individuals affected with NA. Unaffected individuals are not shaded. Diagonal slashes represent deceased individuals. * Indicates individuals with acanthocytosis and the selective alteration of the 4.1R protein of the erythrocyte membrane.

We studied four patients from four different families showing hyperkinetic movement disorders and acanthocytosis. Pedigrees are shown in Fig. 1. The patients provided us with their medical history, then underwent: a neurological examination, psychiatric evaluation, neuropsychological testing, electroencephalogram (EEG), magnetic resonance imaging (MRI) brain scans, electromyography (EMG), electroneurography (NC), fundoscopic examination, cardiological assessment and gave their informed consent to the collection of a blood sample for molecular analysis. The following haematological tests were studied in all patients: liver and thyroid functions, serum electrolytes, serum vitamin A and E levels, ceruloplasmin, haptoglobin, creatine phosphokinase, cholesterols (total, HDL, LDL), triglycerides, and lipoproteins (ApoA1, ApoB). Moreover, the expression of the Kell blood group antigens was evaluated.

To check for the presence of expanded Huntington's disease (HD) alleles, the genetic analysis of the CAG distribution and adjacent polymorphic CCG repeats in the Huntington gene was performed in all of the affected individuals, using previously reported methods [8,9]. All 73 exons plus flanking intronic sequence in the chorein gene were screened for mutations by direct sequencing in four probands with NA, as already described [10-12].

In order to perform membrane protein analysis the erythrocyte membrane proteins were obtained from leukocyte and platelet free red cells lysed with 5 mM Na2HpO4 pH 8.0 containing 0.1 mM PMSF. The electrophoretic analysis of red blood cell (RBC) membrane proteins was performed with 7.5% polyacrylamide gel electrophoresis in sodium dodecyl sulfate (SDS-PAGE) [13]. The ghosts were dissolved to a concentration of 1 mg protein/ml in SDS sample buffer and 0.1 mg was loaded on gel. Crude spectrin was extracted from fresh ghosts in 0.1 mM Na/Na2HPO4, 0.1 mM, 0.1 mM EDTA, 0.1 mM DTT, pH 8.0 at 0°C, and the content of spectrin dimers and tetramers was determined on the supernatant by non-denaturing gel electrophoresis (2% agarose in 4 mM Tris, 0.1 mM EDTA, 1% acetic acid buffer, pH 8.0). Patients, controls, and molecular weight standards were always analysed on the same gel and each sample was evaluated three times. Protein bands were stained with Coomassie Blue and quantified by densitometric analysis using a Fluor S Imager (BIO-RAD) equipped with a MultiAnalyst software package. The content of 4.1 R protein was calculated as a percentage value of total membrane proteins according to Lux SE and Palek J, 1995 [3]. The unpaired Student's *t*-test was applied to compare data from patients (n. 4) and controls (n. 21 healthy blood donors). Results are expressed as means ± standard deviation (SD).

## Case presentation

### Patient IV:1 (RM23)

**The proband **– was a 19 year-old male, with no relevant personal or family history showing mild involuntary orofacial movements and chorea in all four limbs. Psychomotor assessment revealed no cognitive impairment. No psychiatric manifestations were evident and the Beck Depression Inventory Scale proved that there was no concomitant depression. EEG and brain MRI did not reveal any pathological findings. Normal EMG and NC activity was detected. Cardiomyopathy was excluded, fundoscopic examination showed no retinal degeneration, and the expression of the Kell blood group antigens was normal. Haematological parameters were within normal ranges, but a marked presence (95%) of abnormal shaped erythrocytes known as acanthocytes were identified on wet-film preparations. The patient's mother (age at examination: 37 years old) and sister did not show any neurological disorders, while a relevant presence of acanthocytic erythrocytes was observed in peripheral blood (35% and 39% respectively).

A decreased content of 4.1R protein was evident in the patient, his mother, and his sister (Table 1). A marked increase in spectrin dimers (35%, Reference Values < 15%) was apparent in the patient (Figure 3). Self-association of spectrin dimers into tetramers is a critical interaction for membrane structure and function [3]. The percentage of spectrin dimers and tetramers in crude extract reflects their relative distribution in the red cell membrane *in vivo*. Increased percentage of spectrin dimers is indicative of membrane fragility. The presence of a 4.1 R protein defect seems to give rise to the cytoskeleton instability.

The diagnosis of HD was excluded by DNA molecular testing and no disease mutations or single nucleotide polymorphisms (SNPs) were found in the ChAc gene.

### Patient IV:1 (RM16)

**The proband **– was a 72 year-old diabetic woman. She was hospitalized for two unexpected falls and the insidious onset of involuntary right arm movements. She developed a progression of continuous choreic-ballistic movements in all four limbs associated with impaired gait and orofaciolingual dyskinesias. Brain MRI scans showed bilateral putaminal T2 hyper intensities, however EEG did not reveal any pathological findings. The Beck Depression Inventory Scale showed a moderate depressive status. The EMG and NC results show axonal neuropathy. No cardiological anomalies were evident, retinal degeneration was absent, and the expression of the Kell blood group antigens was normal. Haematological parameters were within normal ranges, but numerous acanthocytes (77%) were observed on the peripheral blood film preparations. The patient's daughter showed no evidence of any neurodegenerative disorder, although acanthocytes were present in her blood (31%). Exclusion of Wilson's disease and non-Wilsonian hepatolenticular degeneration were presumed since hepatic copper content via liver biopsy was within the normal range and due to the absence of liver failure.

**Figure 2:**
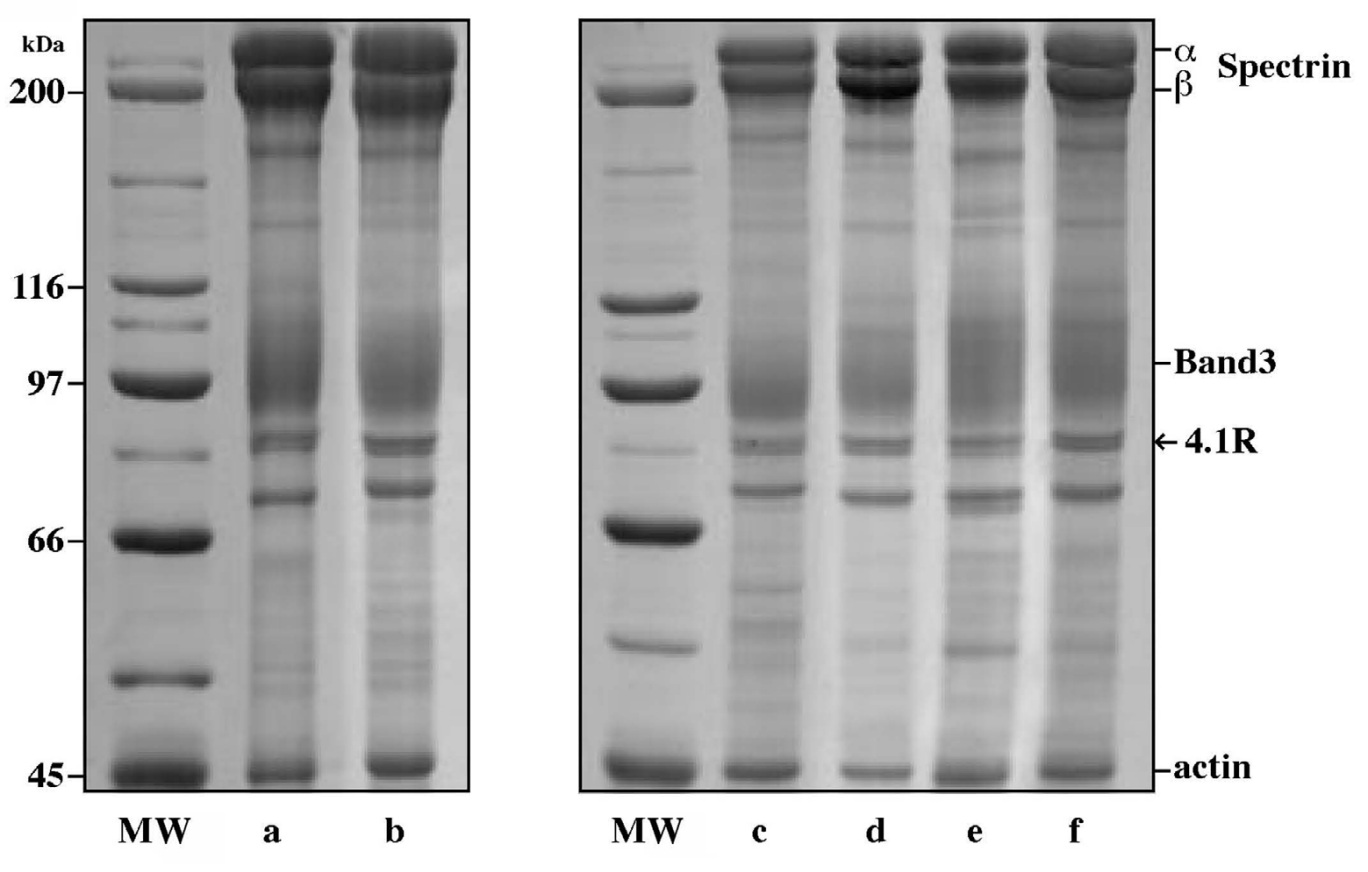
Electrophoretic analyses (SDS-PAGE) of erythrocyte membrane proteins from patients (a = IV:6 RM13; c = IV:1 RM16; d = IV:3 RM15; e = IV:1 RM23) and controls (b, f). MW = molecular weight standards.

**Figure 3:**
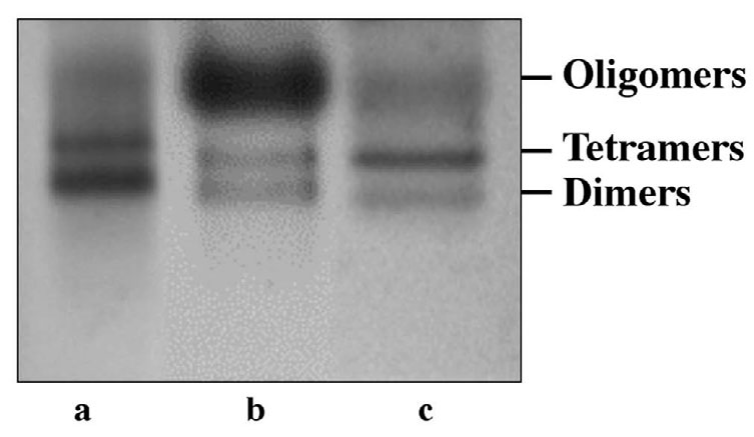
Electrophoretic analyses of crude spectrin extracts from patients (a = IV:1 RM23; b = IV:1 RM16) and control (c).

Analysis of RBC membrane proteins (Fig. 2 and Table 1) showed a decreased content of 4.1R protein and protein band 3 (data not shown), while the spectrin dimers (Fig. 3) were comparable to the control (11%, R.V. < 15%). Therefore the 4.1R defect might affect vertical interactions of skeletal attachment to membrane. Furthermore, the patient's daughter showed the erythrocyte 4.1R defect (Table 1) in spite of the absence of neurological signs.

The genetic analysis of the CAG distribution and adjacent polymorphic CCG repeats in the HD gene was normal and an absence of mutations in the ChAc gene was reported.

### Patient IV:3 (RM15)

**The proband **– was a 65 year-old man suffering from hypertension. He reported a five-year history of depression and orofaciolingual involuntary movements. The depression had been treated with tricyclic drugs and selective serotonine-uptake inhibitors. The neurological examination showed orofaciolingual diskinesias and right distal limb dystonia. The Beck Depression Inventory showed a moderate depressive status. EEG and brain MRI did not reveal any pathological findings. The EMG and NC recordings showed normal electrical activity. Cardiological assessment was normal, retinal degeneration was not present, and the expression of the Kell blood group antigens was normal. Haematological parameters were within normal ranges, although acanthocytes (32%) were observed on the peripheral blood film preparations.

In this patient a decreased content of 4.1R protein was observed (Fig. 2 and Table 1) and an increased amount of spectrin dimers was measured (31%, R.V. < 15%) with an impairment of the dimer self-association into tetramers.

A normal distribution of CAG and adjacent polymorphic CCG repeats in the HD gene was reported and a genetic search for ChAc gene mutations was negative.

### Patient IV:6 (RM13)

**The proband **– was a 38 year-old man who was admitted to hospital because of an isolated generalized tonic-clonic seizure. He reported a four-year history of abnormal involuntary upper limb movements of variable severity. In his case history an acute episode of aggressive behaviour associated with delirium of persecution was also reported. Neurological examination showed ballistic and choreic movements of the arms (left > right) associated with trunk dystonia and orofaciolingual diskinesias. Psychiatric evaluation revealed anxiety, paranoia, depression, obsessive behaviour, and marked emotional instability. EEG and brain MRI did not reveal any pathological findings. Axonal neuropathy was detected by EMG and NC recordings. Cardiomyopathy was not found, fundoscopic examination was normal, the same as the expression of the Kell blood group antigens. Haematological parameters were within normal ranges: however, acanthocytes (85%) were observed on the peripheral blood film preparations.

Hypoxanthine-guanine phosphoribosyltransferase (HPRT) enzyme activity in cells from cultured fibroblasts was normal, excluding adult onset Lesch-Nyhan syndrome. The diagnosis of Gilles de la Tourette syndrome was excluded by reason of onset after 18 years of age and because of the absence of both multiple motor and one or more vocal tics during the illness.

In this patient a decreased content of 4.1R protein was observed (Fig. 2 and Table 1) and an increased amount of spectrin dimers was measured (25%, R.V. < 15%) with an impairment of the dimer self-association into tetramers.

The CAG units and adjacent polymorphic CCG repeats in the HD gene were in the normal range and no disease-causing mutation was found in the ChAc gene.

## Conclusion

In the present study we report, for the first time, four cases of NA showing choreic manifestations and acanthocytosis with a novel alteration in the expression of the erythrocyte membrane proteins: a 4.1R protein deficiency. Structural modification of membrane proteins has rarely been observed in NA; only a few cases of autosomal recessive ChAc with abnormalities of the major integral membrane protein band 3 have been reported [1,14-17]. Moreover, ChAc has never been associated with 4.1R protein alterations.

Acanthocytosis and abnormalities of erythrocyte membrane proteins were revealed in all of the patients. The erythrocyte membrane defect was due to a decreased content of 4.1R protein, a multifunctional skeletal protein necessary for membrane stability and flexibility. The 4.1R protein content was significantly lower in patients (3.40 ± 0.42) than in controls (4.41 ± 0.40, P < 0.0001), reflecting weakened interactions of the cytoskeleton with the membrane. In three patients the 4.1R protein defect involved horizontal cytoskeleton interactions as shown by the increased content of spectrin dimers and was indicative of an impairment of the dimer self-association into tetramers; in a patient with a decrease in protein band 3, the 4.1R protein defect might impair the vertical interactions with integral membrane proteins by affecting the skeletal attachment to membrane.

The discovery of the new specific erythrocyte membrane protein defect, an alteration of 4.1R protein, explains the morphological changes in acanthocytosis and can provide indications regarding the disease protein function. It remains unknown whether genetic defect(s) could be responsible for the two distinct phenotypes: acanthocytosis and hyperkinesia. Interestingly, acanthocytosis and the 4.1R protein defect were also observed in other members of the families showing no neurological symptoms. If phenotypes observed in the families are due to genetic defect(s), one possible explanation could be that unaffected members might have a single mutation with a normal allele, i.e. they might be in a heterozygous state, which leads to the phenotype of acanthocytosis only. Mutations in both alleles or in combination with other genetic modifier(s) would be required for the two distinct phenotypes. Identification of genetic defect(s) in these families would reveal the underlying mechanism(s) of both phenotypes.

None of the patients presented the clinical or biochemical abnormalities typically observed in either abetalipoproteinaemia or McLeod syndrome [1,2].

It is known that 4.1R protein is selectively expressed in haematopoietic tissues and in specific neuronal populations [3]. Erythroid 4.1R protein is an 80-kDa skeletal protein required for structural organization and maintenance of the RBC cytoskeleton [11]. 4.1R protein interacts with spectrin and actin by strengthening the skeletal network and stabilizes the spectrin-actin complexes through the spectrin-actin binding (SAB) domain. Furthermore, the 4.1R N-terminal domain mediates the attachment of the underlying cytoskeleton to the overlying lipid bilayer through interactions with integral membrane proteins such as band 3 and glycophorin C [3]. Abnormalities of 4.1R protein are associated with congenital RBC defects leading to severe membrane fragmentation and hereditary elliptocytosis [3,7].

Four genes encoding 4.1 proteins are known to be expressed in the brain. The corresponding proteins are known as 4.1R, 4.1G, 4.1B, and 4.1N. 4.1 proteins are highly conserved and retain the same fundamental organization of domains [4,5,7,18]. High levels of 4.1R were discretely localized in granule cells in the cerebellum and dentate gyrus [19]. 4.1R protein is also selectively localized in central neurons interacting with the intermediate filament proteins of post-synaptic densities [7,20]. Recently, it has been postulated that 4.1 proteins play an essential role in synaptic plasticity by delivering specific subunits of glutamate AMPA receptors in central synapses [6]. In line with this hypothesis 4.1R protein-null mice, as well as the abnormal morphology and lowered membrane stability of RBC, have specific deficits in movements, coordination, and balance [19], therefore highlighting a connection between 4.1R protein deficiency and human neurodegenerative syndromes.

The present observation suggests a likely association between a defect of 4.1R protein and the expression of hyperkinetic disorders caused by a possible abnormal glutamatergic transmission in the basal ganglia [21]. Pre- and/or post-synaptic modulation of glutamate AMPA receptor-mediated transmission, by facilitating glutamate release from corticostriatal terminals or AMPA receptor currents with ampakines [22,23], might prove beneficial in treating motor disorders associated with 4.1R alterations.

Our sequence analyses did not show any disease-associated mutations or polymorphisms in the ChAc. If the decreased expression of 4.1R protein is due to genetic defect(s), two possible explanations might be considered for the results of our genetic study. Firstly, there might be other genetic defect(s) in the ChAc gene including the regulatory region, which was not examined in our analysis. Secondly, there might be further as yet unknown genetic defect(s) or further locus heterogeneity in NA. However, previous papers have revealed little evidence of further locus heterogeneity in neuroacanthocytosis [10].

We have demonstrated that expression of 4.1R protein in erythrocytes is decreased in patients with atypical neuroacanthocytosis, but there is no evidence of decreased expression of the protein in the brain because we did not examine post-mortem brains of patients. Analyses of 4.1R-deficient mice showed lower membrane stability of RBC, movement abnormalities, abnormal morphology and decreased expression of the protein in the brain nucleus/tissues including granule cells and dentate gyrus [24]. The functional effect(s) of decreased expression of 4.1R protein in the brain remains unknown. A previous study revealed that 4.1R proteins play an essential role in synaptic plasticity by delivering specific subunits of glutamate AMPA receptors into central synapses [25]. The altered metabolism of glutamate AMPA might lead to dysfunction of neurotransmission in the basal ganglia and hyperkinetic movements in neuroacanthocytosis.

It also remains unknown whether the expression pattern of protein in erythrocytes might reflect their expression patterns in the brain. An investigation into the expression of alpha-synuclein in lymphocytes from PARK1-patients demonstrated that decreased expression of alpha-synuclein correlates with the severity of the clinical phenotype [26], suggesting that expression patterns of protein in peripheral tissue might not be independent from those in the central nervous system. Investigation of expression of 4.1R protein in post-mortem brains of patients with neuroacanthocytosis is a prerequisite for validation of decreased expression of 4.1R protein and understanding biological mechanism(s) for hyperkinesia in NA.

In conclusion, our study demonstrates decreased expression of 4.1R protein in the erythrocytes, which might reflect decreased expression of this protein in the brain resulting in hyperkinetic movements in neuroacanthocytosis.

## Abbreviations

CERC = Centro Europeo di Ricerca sul Cervello; IRCCS = Istituto di Ricovero e Cura a Caratttere Scientifico; NA = Neuroacanthocytosis; ChAc = Chorea-acanthocytosis; HD = Huntington's disease; HPRT = Hypoxanthine-guanine phosphoribosyltransferase; RBC = Red blood cell; R.V. = Reference Values; SDS-PAGE = Polyacrylamide gel electrophoresis in sodium dodecyl sulphate; SD = standard deviation; SNPs = single nucleotide polymorphisms; SAB = spectrin-actin binding.

## Competing interests

The author(s) declare that they have no competing interests.

## Authors' contributions

AO, PCal, and PCap made significant contributions to conception and design, acquisition of data and drafting the manuscript; TK and PCi revised the study critically for important intellectual content; AR and GB collected the clinical data set; AT and AMS participated in the experimental biochemistry work and analysis of data; AS and AP were involved in the interpretation of results and general conclusions. All of the authors read and approved the final manuscript.

## Pre-publication history

The pre-publication history for this paper can be accessed here:



## Figures and Tables

**Table 1:** Densitometric analysis of the erythrocyte 4.1R protein

Index of patients	4.1R (%)*
IV:1 (RM23)	3.8 ± 0.10
IV :1 (RM16)	2.8 ± 0.35
IV :3 (RM15)	3.3 ± 0.20
IV:6 (RM13)	3.6 ± 0.25

Relatives	

IV:2 (RM23)	3.2 ± 0.30
III:5 (RM23)	3.1 ± 0.20
V:2 (RM16)	3.0 ± 0.30

Controls (n = 21)	4.41 ± 0.40^

* Patient values expressed as mean ± SD (n = 3)

^Reference values expressed as mean ± SD; from n.21 healthy blood donors. The 4.1R content was considered low when it was below the reference values.
